# Assessment of biocompatibility of 3D printed photopolymers using zebrafish embryo toxicity assays†
†Electronic supplementary information (ESI) available: Supporting Fig. S1–2 and Table T1. See DOI: 10.1039/c5lc01374g

‡
‡We would also want to draw the attention of the reader to the availability of the dataset associated with this paper, available here (http://dx.doi.org/10.5525/gla.researchdata.238).


**DOI:** 10.1039/c5lc01374g

**Published:** 2015-12-08

**Authors:** N. P. Macdonald, F. Zhu, C. J. Hall, J. Reboud, P. S. Crosier, E. E. Patton, D. Wlodkowic, J. M. Cooper

**Affiliations:** a Division of Biomedical Engineering , School of Engineering , University of Glasgow , Rankine Building , Oakfield Avenue , Glasgow G12 8LT , UK . Email: Jon.Cooper@glasgow.ac.uk; b The BioMEMS Research Group , School of Applied Sciences , RMIT University , Melbourne VIC 3083 , Australia; c Department of Molecular Medicine and Pathology , University of Auckland , Auckland 1023 , New Zealand; d MRC Institute of Genetics and Molecular Medicine , MRC Human Genetics Unit , Edinburgh , UK

## Abstract

3D printing has emerged as a rapid and cost-efficient manufacturing technique to enable the fabrication of bespoke, complex prototypes. If the technology is to have a significant impact in biomedical applications, such as drug discovery and molecular diagnostics, the devices produced must be biologically compatible to enable their use with established reference assays and protocols. In this work we demonstrate that we can adapt the Fish Embryo Test (FET) as a new method to quantify the toxicity of 3D printed microfluidic devices. We assessed the biocompatibility of four commercially available 3D printing polymers (VisiJetCrystal EX200, Watershed 11122XC, Fototec SLA 7150 Clear and ABSplus P-430), through the observation of key developmental markers in the developing zebrafish embryos. Results show all of the photopolymers to be highly toxic to the embryos, resulting in fatality, although we do demonstrate that post-printing treatment of Fototec 7150 makes it suitable for zebrafish culture within the FET.

## Abstract 2

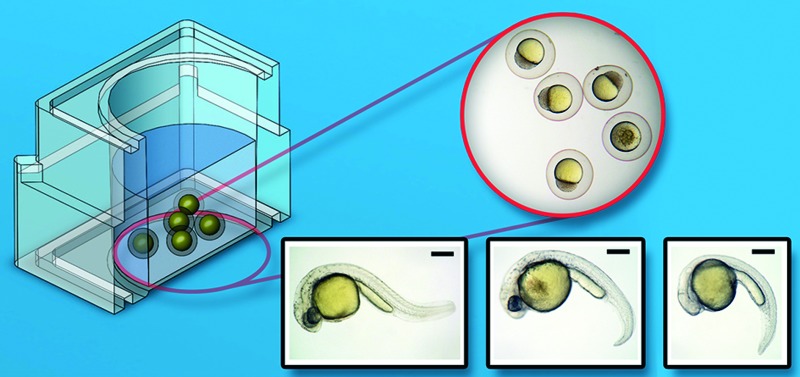
3D printing enables the rapid and cost-efficient manufacturing of bespoke, complex prototypes. We show that biocompatibility needs to be considered carefully and provide a specific assay to that effect.

## Introduction

3D printing is a rapid prototyping process technology, whose popularity has been growing, allowing researchers to generate physical parts or devices in a short period of time (hours or days), directly from computer-based designs.^1^ Within biomedical engineering, these methods have been applied to the fabrication of microfluidic devices,^2–5^ medical imaging^6^ and scaffolds for living cells.^7^ Fabrication of microstructured scaffolds for cell assays using rapid prototyping has also been investigated using associated techniques including selective laser sintering,^8^ layered hydrospinning,^9^ laser stereolithography (SLA),^10^ digital light projection,^11^ and two-photon lithography.^12^ Bespoke 3D printing of biodegradable and biocompatible materials has also emerged as a new fabrication technology, but generally requires specific processes, each tailored to a given application.^13^

Most recently, the practical limitations imposed by the use of opaque 3D printing materials in applications involving imaging have been overcome by optimising the chemistry of the resins used to create micro-^5^ and milli-fluidic platforms.^14^ Biocompatibility of commercially-available opaque materials in combination with membranes has been demonstrated using a cell based assay for inkjet printing of polymers.^15^ It is however becoming widely acknowledged that more detailed biocompatibility testing is needed.^16,17^

3D printing is now recognised as providing rapid turnaround within a narrow design space, enabling the production of devices on demand,^18–20^ for applications such as personalised medicine and therapy monitoring. In some cases it has been shown to be more cost-effective than standard soft lithography^21^ (for example stereolithography has enabled the manufacturing of complex fluidic structures such as microvalves,^22^ whilst others have demonstrated the capabilities of the technique to create libraries of devices).^23^

Here we adapted the established zebrafish FET test to provide a new method to investigate the biocompatibility of photopolymers used in commercial 3D printers to create assay structures to culture zebrafish embryos. The zebrafish FET provides an established vertebrate model (*e.g.* OECD test for chemicals #236^24^) that been extensively used to study human diseases and genetics, both in the laboratory and within microfluidic systems.^25–28^ In our paper we now propose to use the zebrafish as a proxy to indicate the toxicity of the 3D printing polymers (and associated solvents). We also show that we can use the test to optimise post-treatment processing of 3D printed structures.

**Fig. 1:**
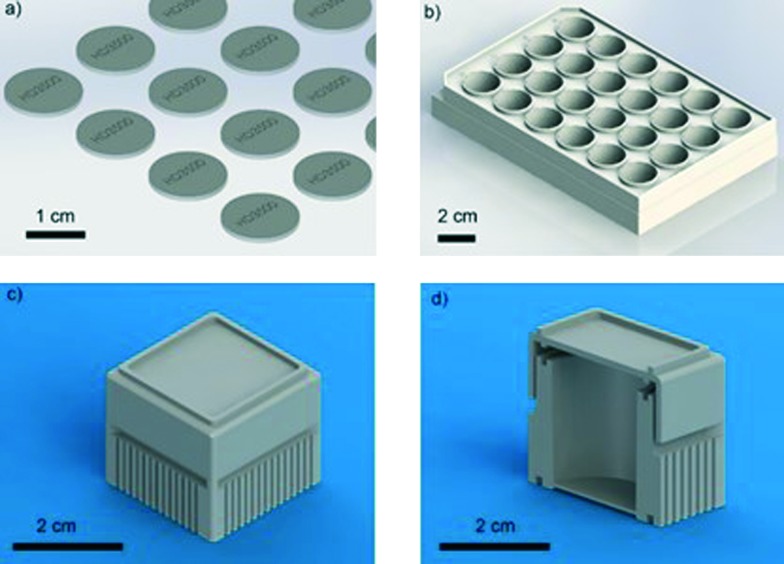
3D rendered images of designs used for biocompatibility of 3D printed materials with zebrafish embryos. (a) Discs for inserting into a 24-well plate, engraved (15 mm in diameter, 1 mm thick). (b) 24-well plate based on flat-bottomed polystyrene plates available commercially. (c) Single-well culture device with lid. (d) Cross-section of single well culture device. Scale bars are 1 and 2 cm respectively.

We chose to study four different photopolymers, some of which, such as VisiJet Crystal EX200, are United States Pharmacopeia (USP) Class VI certified materials (the strictest class for plastic biocompatibility) and in principle safe; others (such as Watershed and Fototec 7150) have been less well characterised. We investigated the development of the FET system, using 3D printed standard 24-well plate designs (Fig. 1), to optimize and validate treatment of the commercially available polymers chosen (VisiJet Crystal, S300 Support Wax, Watershed, Fototec 7150, ABS – see Table S1† for material details). We assessed the viability and development of zebrafish embryos over extended culture.

It is worthy to note that, in the context of testing materials for Lab-on-a-Chip and microsystems applications, as the device dimensions decrease, the surface area to volume ratio increases. The consequence of this is that any biological organism or entity (including cells or biomolecules), contained within the printed structure, is exposed to a greater amount of the printed material (and/or solvent contained therein), relative to the case for larger structures. Thus, the 3D printer polymer's biocompatibility becomes increasingly significant as device geometries shrink, within miniaturised systems.

## Experimental section

### 3D printing materials

VisiJetCrystal EX200, (along with its support material VisiJet S300), Watershed 11122XC, and Dreve Fototec SLA 7150 Clear were selected due to their transparency and potential use in imaging. ABSplus P-430 was selected to represent fused deposition modelling (FDM) fabrication methods.

VisiJet® Crystal and VisiJet® S300 Support Material was supplied by 3D Systems, Australia. WaterShed XC 11122 was supplied by Somos, Australia. Dreve Fototec 7150 Clear was supplied by Dreve Otoplastik GmbH, Germany. The composition of these materials is proprietary, however they are typically based upon acrylate monomers (available information on contents can be found in Table S2 in ESI†). Acrylonitrile butadiene styrene (ABS) 655 cm^3^ rolls were obtained from Solutions 2 Enterprise, UK. SLA, inkjet^29^ and digital processing SLA (DP-SLA)^5^ using photopolymers provide the highest resolutions for microfluidic applications. Further information regarding 3D printer materials used in biological applications for microfluidics is available in the literature.^17^

### 3D printers and fabrication

In this work three rapid prototyping machines were used. The Hewlett-Packard (HP) DesignJet, 3D Systems HD3500 Plus and Viper Pro (Plastic Design Technology, NZ) additive manufacturing machines, see Table 1. Unless stated otherwise, all samples used in this study were fabricated following the guidelines set by the manufacturer.

**Table 1:** List of additive manufacturing machines used in this work with main specifications

Machine (manufacturer)	Technology (print mode)	Accuracy/layer thickness (μm)	Material
HD3500+ (3D Systems)	MJM (HD)	50/32	VisiJet Crystal (USP Class VI), VisiJet S300
	MJM (UHD)	25/29	
	MJM (XHD)	25/16	
Viper Pro (3D Systems)	SLA	25/50	Watershed 11122XC Dreve Fototec 7150 Clear
DesignJet	FDM	120/254	ABS

Samples printed using the DesignJet HP were directly printed onto the bed as no support was required. Viper Pro samples printed using the Viper Pro system; samples were washed in isopropanol followed by water and then air dried. Printed HD3500+ samples were placed in an oven (Thermotec 2000, Contherm, NZ) at 70 °C causing the wax support to melt. Following this, samples were rinsed in warm (50 °C) soapy water. For optimal transparency, samples were placed in an oven at 60 °C for 10 minutes. The oven was then switched off and allowed to cool for 1 hour allowing the models to cool slowly. If cooled rapidly the thermo-polymer VisiJet Crystal forms misty, crack like defects impairing the optical quality.

### Design

As stated, three different experimental systems were used to test the biocompatibility of the polymers, namely (a) discs for inserting into a 24-well plate, engraved here with HD3500 label (15 mm in diameter, 1 mm thick); (b) 24-well plate based on flat-bottomed Corning plates; and (c) bespoke single-well culture device with lids (Fig. 1).

For measurements involving discs, samples were printed using both the VisiJet Crystal and Watershed polymers for testing of biocompatibility with zebrafish embryos, Fig. 1(a). The single well culture system shown in Fig. 1(c and d) was printed using the HD3500+, Viper Pro (Watershed + Dreve) and HP DesignJet. The design was based on the commercially available Corning 24-well plate dimensions shown in Fig. 1(b). The lid fits loosely to allow the diffusion of gases into the culture-well but securely enough not to be dislodged during handling. The disc samples printed using HD3500+ and Viper were diluted in 1 litre of DI water and stirred using a stir bar for a period of 24 hours; this process was used for the single wells as well.

### Zebrafish husbandry and embryo culture

Wild-type zebrafish Danio Rerio (AB line) and transgenic Tg (fli1a:EGFP) adult zebrafish were obtained from the Zebrafish International Resource Center (Oregon, Eugene, OR, USA) at the University of Auckland School of Medicine, Auckland, New Zealand.^30–32^ Wild type zebrafish were also used at the Medical Research Council Human Genetics Unit, Edinburgh, UK. Adult zebrafish were kept in a 14 hours light, 10 hours dark cycle fish facility and fed twice daily with artemia and once daily with dry feed. Zebrafish embryos were obtained from random pair-wise mating and natural spawning. Collected embryos were maintained in embryo medium E3 and rinsed to remove debris and dead embryos. Embryos were cultured at the optimal temperature of 28.5 ± 0.5 °C in E3 medium and developmentally staged as described earlier^33,34^ as well as during microfluidic culture experiments. Animal research was conducted with approval from The University of Auckland Animal Ethics Committee (approval ID R661/1) and University of Edinburgh Ethics Committee respectively.

### Embryo culture, treatment and phenotype analysis

The zebrafish embryos were obtained from random pair-wise mating using the marbling technique.^35^ Embryos were collected, dead/unfertilised embryos and debris were removed by pipetting. Embryos of 1.5 hpf and/or 24 hpf were chosen for experimentation. Viability was observed by checking: (i) lethal endpoints (cumulative mortality): coagulation, lack of tail detachment, lack of somite formation; (ii) sub-lethal developmental endpoints: development of eyes, spontaneous movement, heartbeat and blood circulation, pigmentation, formation of edemata; (iii) endpoints of teratogenicity: malformations of head/face/arches/jaw general retardation.^36–38^ The potential of hatching and time to hatch was also considered.^36,37^ Temporary anaesthesia to inhibit intrinsic movements during fluorescent imaging was obtained by adding tricanine mesylate (0.2 mg ml^–1^) 15 minutes before image acquisition. Post experiment, all hatched zebrafish were euthanized at –20 °C.

### Imaging and data analysis of zebrafish

General stereoscopic images of embryos grown on chip-based devices were obtained using the Leica MZ7.5 stereomicroscope from Leica Microsystems, Wetzlar, Germany. Stereoscopic fluorescence images were taken using a Nikon SMZ1500 from Nikon, Japan. Stereoscopic images were collected with a Nikon SMZ1500 and Nikon E5400 Coolpix camera from Nikon, Japan. Data analysis of collected images was completed using the Leica Application Suite (LAS) (Leica Microsystems) and ImageJ.

For images of Tg (fli1a:EGFP) zebrafish, a Nikon SMZ1500 (Nikon, Tokyo, Japan) connected to a DS-U2/L2 camera was used. Fluorescent images were also taken using an AM4113T-GFBW Dino-Lite Premier USB microscope. Imaging of printed devices was captured using a Leica MZ7.5 stereomicroscope equipped with a Leica DFC295 CMOS camera running under the LAS Multitime software (Leica Microsystems, Wetzlar, Germany). For biocompatibility experiments, the zebrafish were imaged using a Nikon SMZ1500 (Nikon, Tokyo, Japan) with a Nikon E5400 Coolpix camera.

### Data analysis and controls

Data analysis and presentation was performed using LAS (Leica Microsystems); ImageJ; Microsoft Excel 2010; SolidWorks 2012 (Dassault Systèmes SolidWorks Corp) and SolidView 2012 (Stratasys Direct Manufacturing).

## Results and discussion

3D printed discs shown in Fig. 1(a) were first fabricated in the five polymers (VisiJetCrystal EX200, VisiJet S300, Watershed 11122XC, Fototec SL.A 7150 Clear and ABSplus P-430, as listed in Table 1) and placed at the bottom of standard plastic 24-well plates along with standard Corning 24-well plates as positive controls of biocompatibility. We performed a long-term culture of wild-type AB zebrafish embryos (1.5 hpf), with each well having 5 embryos placed inside in standard culture conditions of 1 ml of E3 media, at 28.5 °C over 90 hours.

**Fig. 2:**
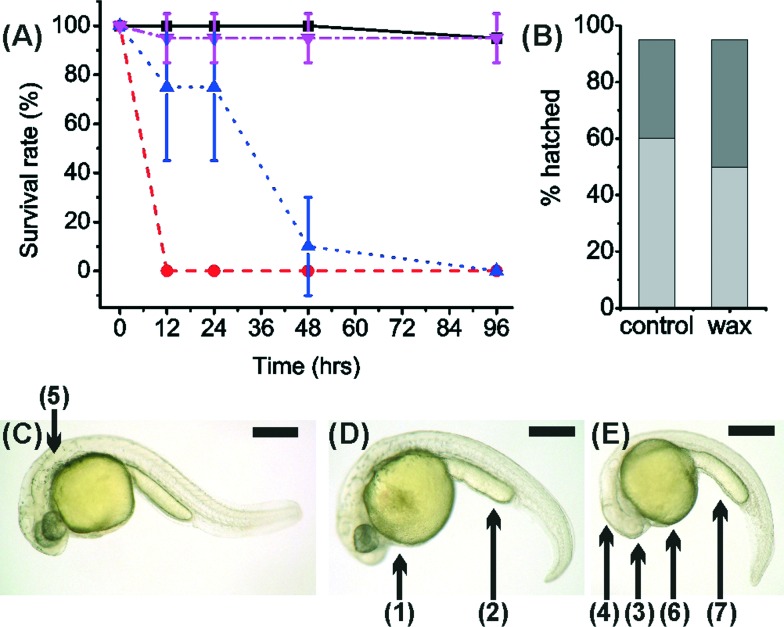
(A) Survival rate of zebrafish embryos in 3D printed material VisiJet Crystal without S300 support material (red circles) and with (dark blue triangle). Zebrafish cultured within a Petri dish containing S300 (magenta inverted triangle) and without as a control (black square). Error bars span one standard deviation from the mean (B) hatching of zebrafish embryos at 48 h (light grey) and 96 h (dark grey), in a Petri dish containing S300 (‘wax’) and without (‘control’). (5 embryos per well, *n* = 5). (C–E) Morphology analysis at 27 hours incubation of zebrafish with 3D printed materials. Stereomicroscopy images of dechorionated zebrafish embryos immobilised in agar: (C) control zebrafish embryo cultured in a 24-well plate development is normal. (D) Image of zebrafish cultured with Watershed material. Development has been stunted, appears 2–3 hours behind control, darkening of yolk sac (1) indicating toxicity as well as roughing and widening yolk extension (2). (E) Image of zebrafish cultured on VisiJet Crystal. Development has been stunted by *ca.* 5 hours. Eyes (3) and brain (4) have not developed, pigmentation (shown as (5) in C) is not present, unusual yolk sac (6) and yolk extension (7) shape, all show retardation. Scale bar is 500 μm.

We also performed long-term culture of the wild-type zebrafish within 3D printed wells – designed according to the 24-well plate dishes used above. These were also fabricated with and without wax support material. Control samples were melted wax support material (10 mg) at the bottom of a standard Corning 24-well plate and clean untreated Corning wells. The development of zebrafish within a 3D printed well without wax was entirely unsuccessful and all embryos were dead by 19 hours Fig. 2(A).

It was also observed that *ca.* 70% of zebrafish embryos within 3D printed wells with wax developed after 24 hours. By 48 hours, the survival rate was *ca.* 10% and all were dead after 90 hours. Fish grown in samples containing wax and in control samples were all observed to have normal uniform development Fig. 2(B), showing that the high toxicity observed was not due to the wax support material. In fact, the presence of wax support in the 3D printed wells deferred death by *ca.* 40 hours (but did not stop death) a fact that can be attributed to the wax support material acting as an additional barrier to the diffusion of any toxic material into the E3 medium.

Incubated zebrafish in the Watershed polymer were stunted compared to the control sample (2.86 ± 0.13 mm) Fig. 2(C), with typical length of 2.63 ± 0.34 mm. Although eyes and pigmentation were present, a darkening of the yolk sac, indicative of toxicity, and enlarged yolk extension were observed Fig. 2(D). Stunted growth (2.57 ± 0.25 mm) was also observed in VisiJet Crystal incubated samples Fig. 2(D), where eyes and pigmentation were missing, yolk sac and extensions have abnormal shapes and also appear darker.

One commonly used definition of biocompatibility is “the ability of a material to perform with an appropriate host response in a specific application,”^39^ which highlights the importance of the context of use and careful consideration when comparing data and conclusions from different experiments. Thus whilst VisiJet Crystal material is classified as biocompatible for medical devices, this clearly does not translate into suitability for zebrafish developmental studies, which act as proxies to show the acute toxicity of the material. In this context, it should be noted that zebrafish sometimes respond fatally where doses would be harmless to humans (for example synthetic detergents in doses between 0.4 and 40 mg l^–1^ are highly lethal to zebrafish).^40^

Notwithstanding this, the zebrafish FET now forms the basis of an OECD standard test for chemical risk assessments, which uses the development of embryos in wellplates over 96 hours as a measure of acute toxicity.^24^ The test uses developmental defects such as coagulation, lack of somite and heartbeat as well as non-detachment of the tail, as endpoints. USP Class VI tests were primarily designed to evaluate plastics for pharmaceutical packaging, where three tests are performed over a 5 day period: systemic injection, intracutaneous testing and implantation tests; all of which are performed on animals. There is a complete list of tests and further details regarding the FDA regulations on medical devices.^41^ At the time of writing, VisiJet Crystal has not passed the ISO 10993.

The hypothesis that one or more toxic compounds leached from the 3D printed parts was tested by washing the discs within a large volume of solvent under agitation, after manufacture and before testing. To explore this, two assays were used: a semi-quantitative longitudinal study, recording established zebrafish developmental features, size and survival over 48 hours for two different aged embryos (1.5 and 24 hpf); and a second assay, specifically looking at rates of hatching of eggs.

**Fig. 3:**
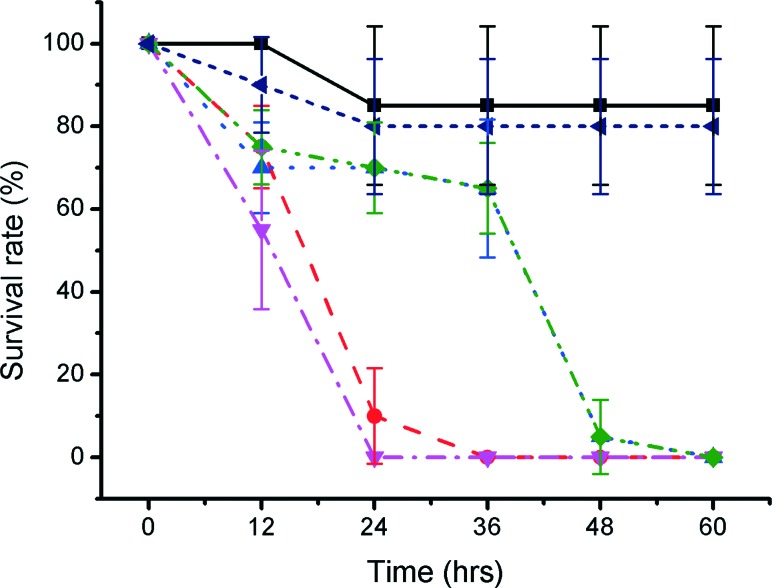
Graph showing survival of zebrafish cultured with 3D printed materials. Zebrafish at 1.5 hpf were incubated (5 embryos per well, *n* = 5) with VisiJet Crystal (red circles) and Watershed (magenta inverted triangles) and ABS (violet arrow) samples. Additionally, DI washed VisiJet Crystal (blue triangle), DI washed Watershed (green diamond) and Petri dish cultured samples (black square) are shown. Error bars span one standard deviation from the mean.

In the first assay, after 12 hours, the survival rate of 1.5 hpf embryos cultured with washed VisiJet Crystal discs (7% ± 10%) was comparable with that of unwashed VisiJet Crystal discs (75% ± 10%). The washing procedure provided a limited improvement for Watershed washed samples (75% ± 9% survival compared to 55% ± 19%). The survival in unwashed samples of both materials continued to decrease after *ca.* 24 h (0% for Watershed and 10% ± 11% for VisiJet). In general, washing maintained some survival up to *ca.* 24 h but by *ca.* 48 h resulted in fatality for VisiJet Crystal (viability was only 5% ± 9%). Survival rates for control samples at comparable times were 85% ± 19%, Fig. 3.

**Fig. 4:**
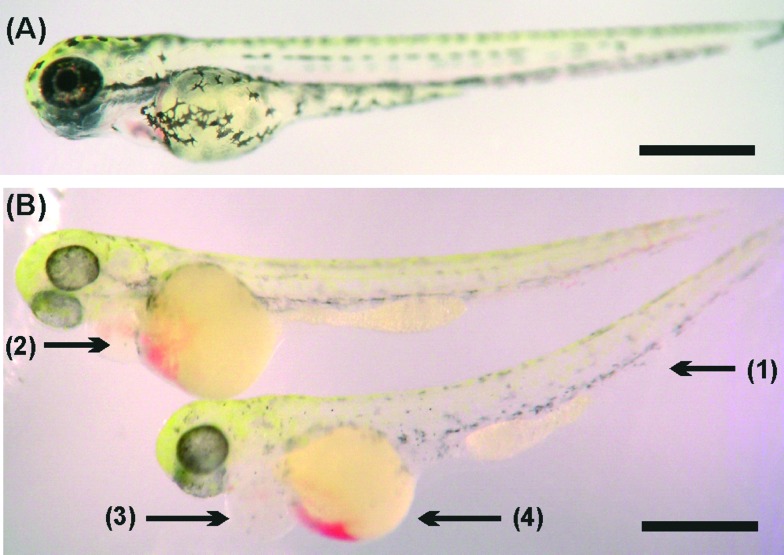
Morphological changes during incubation with 3D printed materials of 72 hpf zebrafish: a) stereomicrograph of hatched zebrafish embryo control showing normal morphology development. b) Zebrafish after 48 hours of incubation with washed VisiJet Crystal samples. These fish are grossly normal, however they show signs of developmental delay as well as hypopigmentation (1), heart edema (2), bloody pooling (3) and reduced yolk extensions (4). Scale bars are 500 μm.

To gain further insights on the effects of the 3D printed materials on the zebrafish embryo development and survival rate, we repeated the study with 24 hpf embryos (Fig. 4, quantified in Fig. S1 in ESI†). The hatched larva appeared to have developed normally but more slowly than the fish incubated in control samples, as indicated by the lower count of melanocytes. However, there was significant bleeding in the yolk sac, which was also observed in the head area. The heart area was seen to be swollen and enlarged compared to the control; these characteristics are not representative of healthy zebrafish embryo development.^33^

Only limited research has been completed on the toxicity of 3D materials to aquatic organisms. Studies on human tissues have highlighted that photopolymerization initiators left in the materials post-production could be implicated in their toxicity. For example, in two-photon 3D printing, the modification of the photoinitiators with a natural compound (riboflavin) has dramatically increased biocompatibility.^42^ Ultra-fine particles (UFP), small, nanosized particles less than 100 nm in diameter, have also been shown to be released from polylactic acid (PLA) and ABS materials, commonly used in low-resolution 3D printers.^43^ The toxicity of nanoparticles has been studied on zebrafish, including silver,^44^ titanium dioxide and zinc oxide.^45^ In human health, UFP are a serious health concern because they deposit efficiently in both the pulmonary and alveolar regions of the lung.^46^ Both chemical composition and particle size have an effect on which cell type is affected and show size-dependent activity.^44^ Condensation of synthetic organic vapours from the thermoplastic feedstock could also be a contributor to toxicity.^43^

One limitation of this study on zebrafish is the restricted information available on the exact chemical composition of the 3D printed materials. Partial composition information can be obtained from the Material Safety Data Sheet of these materials and these are referenced in Table T1 in ESI.† Biocompatibility in relation to chemical composition of materials, particle size and coagulation will require future studies.

**Fig. 5:**
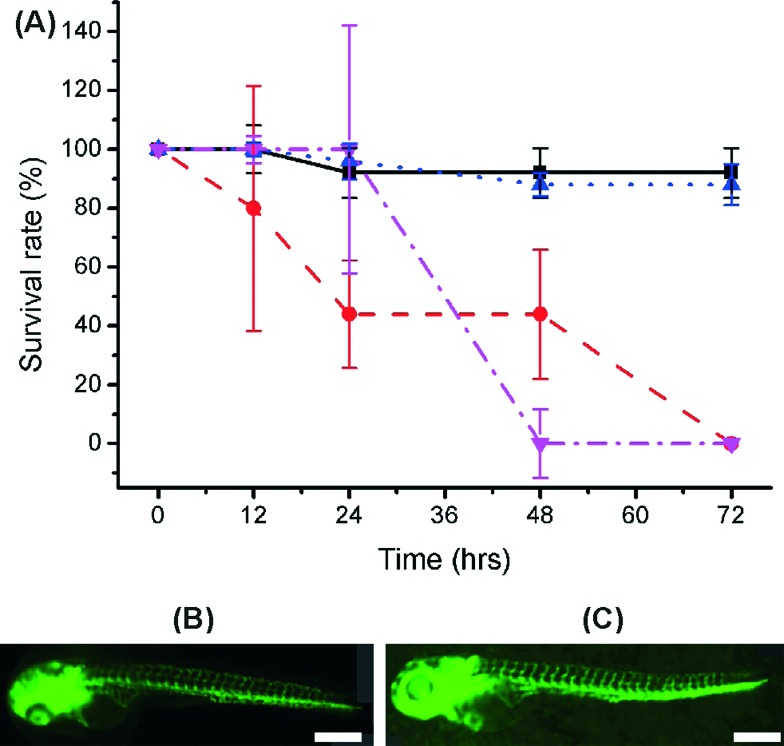
(A) Graph showing the survival rate of embryos cultured over 72 hours with 99% EtoH soaked wells printed in both VisiJet Crystal (red circle) and Fototec 7150 (blue triangle). Untreated Fototec 7150 material (magenta inverted triangle) and Petri dish cultured zebrafish (black square) are also shown (number of embryos was 5, *n* = 3). Error bars span one standard deviation from the mean. Intersegment vessels (ISV) analysis morphology on hatched zebrafish larvae cultured with (B) control petri dish and (C) Fototec 7150 printed wells. Scale bar is 500 μm.

We also explored the use of organic solvents to wash the fabricated parts (Fig. S2 in ESI).† 70% ethanol, 99% ethanol and 99% isopropanol have previously been used to wash away large non-aesthetical debris and support materials (see for example protocols by Formlabs^47^). In our studies, treatment with 99% ethanol significantly improved the outcome for the embryos for some materials and could lead to a practical way to improve biocompatibility. While VisiJet Crystal still exhibited high toxicity (100% mortality occurred at 72 hours – Fig. 5), in Fototec 7150 polymer printed wells, washed with ethanol, embryos developed normally, had comparable viability (88% ± 7%), while unwashed wells were highly toxic.

Intersegment vessel (ISV) morphology analysis on transgenic zebrafish embryos and larvae (fli1a:EGFP, expressing enhanced green fluorescent protein (EGFP) in blood vessels throughout embryogenesis) showed that larvae hatched within Fototec 7150 3D printed wells soaked in 99% ethanol and had normal ISV development compared with larvae hatched from control petri dish Fig. 5.

## Conclusions

In summary, we developed a method to evaluate biocompatibility of 3D printed devices by assessing the viability and development of zebrafish embryos over extended culture and conclude that many of the untreated photopolymers used in commercial 3D printers are unsafe for zebrafish culture. This is surprising as, for example, VisiJet Crystal is a USP Class VI certified material, which has been assessed for use in animals and was expected to show favourable biocompatibility.

We also showed that pre-treatment of Fototec 7150 improves its compatibility with zebrafish culture, making it suitable for fabricating microfluidic devices for biological applications. In conclusion while 3D printing of microfluidic devices is enabling a quickly growing number of applications, our results suggest that caution should be exercised in assessing potential biocompatibility issues. We also provide a route towards the treatment of the fabricated devices with the potential to sustain zebrafish embryos development.
