# Supplementary material for: Assessment of biocompatibility of 3D printed photopolymers using zebrafish embryo toxicity assays
Source: Lab Chip. 2015 Dec 8;16(2):291–7. doi: 10.1039/c5lc01374g (PMC4758231; doi:10.1039/c5lc01374g)
Supplement: Supplementary file 1 [file LC-016-C5LC01374G-s001.pdf]

Electronic Supplementary Material (ESI) for Lab on a Chip.
This journal is © The Royal Society of Chemistry 2015

Assessment of Biocompatibility of 3D printed Photopolymers using Zebrafish Embryo Toxicity Assays

N. P. Macdonald, F. Zhu, C. J. Hall, J. Reboud, P. S. Crosier, E. Patton, D. Wlodkowic and J. M. Cooper

Supplementary Information

Table S1. List of additive manufacturing materials used in this work with main specifications.

Material	Description/ Manufacturer	Composition	Optical Quality	Toxicity	Certifications
VisiJetCrystal EX200	Photopolymer / 3D Systems	Urethane acrylate oligomers 20-40%<br>Ethoxylated bisphenol A diacrylate 15-35%<br>Tripropyleneglycol diacrylate 1.5-3%	Transparent	Urethane acrylate oligomers >2g/Kg NA NA<br>Ethoxylated bisphenol A<br>diacrylate NA Tripropyleneglycol diacrylate > 2g/kg	USP Class VI
VisiJet S300	Wax / 3D Systems	Hydroxylated wax 60 – 100%	Opaque, cream	Oral LD50: 20 g/kg (rat)	None
Watershed 11122XC	Photopolymer/ DSM Corp	N/A	Clear Transparent	N/A	None
Fototec SL.A 7150 Clear	Photopolymer/<br>Dreve Otoplastik	Alkoxilated bisphenol-A-dimethacrylates<br>Urethane dimethacrylate<br>Butanediole Dimethacrylate	Clear Transparent	N/A	None
ABSplus P-430	Thermo Plastic Polymer/ Stratasys	Butadiene-styrene-acrylonitrile-methyl methacrylate copolymer 70-75%<br>Styrene/acrylonitrile copolymer (SAN)<br>25-30%	Opaque, Ivory	Oral LD50 > 5g/kg (rat)<br>Dermal LD50 > 2g/kg (rabbit)	None

LC50: Lethal concentration, 50 percent

LD50: Lethal dose, 50 percent

USP: United States Pharmacopeia

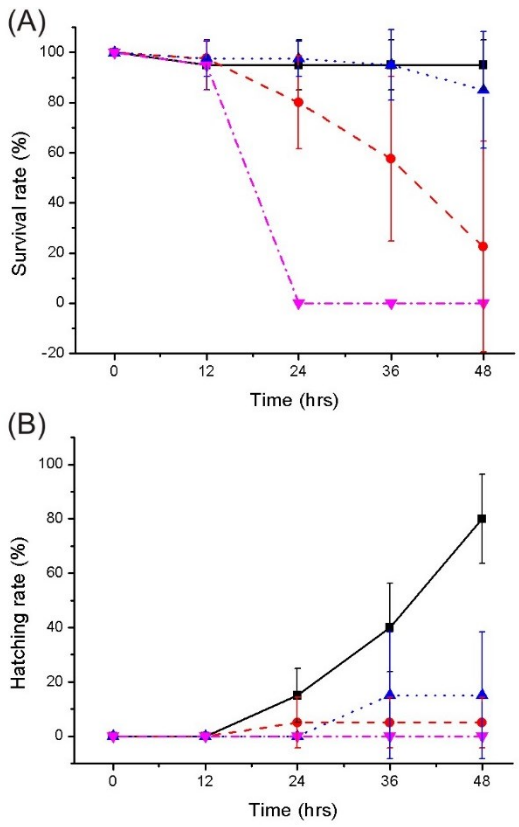

Fig. S1 Graph showing survival of 24 hpf zebrafish cultured with 3D printed materials. Zebrafish at 24 hpf were incubated (5 embryos per well, *n*=5) with VisiJet Crystal (red circles) and Watershed (magenta inverted triangles). Additionally, DI washed VisiJet Crystal (blue triangle) and Petri dish cultured samples (black square) are shown. Error bars span one standard deviation from the mean. (A) Cumulative survival rate of zebrafish over a 48 hour incubation period. Watershed unwashed samples caused cell death within 25 hours of incubation. VisiJet Crystal controls shows a steady reduction of survival, washed samples however have increased survival. (B) Cumulative hatching success of zebrafish embryos over a 48 hour incubation period. Watershed unwashed hatching rate was 0%. Washing of VisiJet Crystal samples improved hatching success to 15% ±16% compared to 5% ± 9% for unwashed samples. Hatching of control samples was 95% ±10%. (n=4). Watershed samples were equally toxic to zebrafish embryos, such that at ca. 25 hours, all zebrafish embryos were dead. In contrast, embryos on washed VisiJet Crystal samples survived longer than younger ones (85% of the embryos on the washed samples remained alive after 48h, a value comparable to the control), while the hatching rate did not improve compared to that of younger embryos.

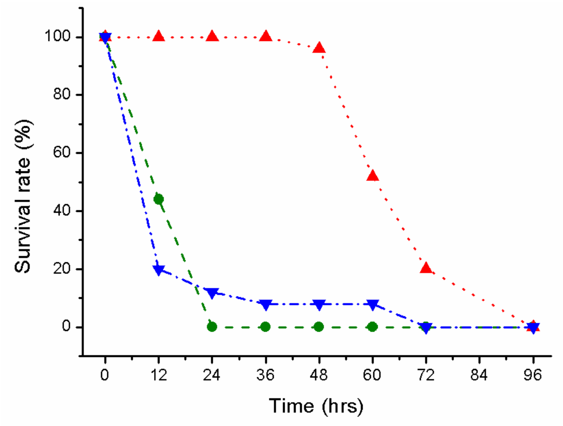

Fig. S2 Graph showing survival rate of zebrafish embryos cultured within VisiJet Crystal wells treated with organic solvents, 5 embryos per well. The wells were treated with organic solvents, 70% EtOH (green circle), 99% EtOH (red triangle), 99% IPA (blue inverted triangle) and compared at Petri dish cultured embryos (black square). We observed that 99% EtOH promoted the highest survival rate of up to 91 hours; embryos cultured with 70% EtOH and 99% IPA treated wells survived for up to 66 hours.
